# Loss of Villin Immunoexpression in Colorectal Carcinoma Is Associated with Poor Differentiation and Survival

**DOI:** 10.1155/2013/679724

**Published:** 2013-09-05

**Authors:** Jaudah Al-Maghrabi, Wafaey Gomaa, Abdelbaset Buhmeida, Mohmmad Al-Qahtani, Mahmoud Al-Ahwal

**Affiliations:** ^1^Scientific Chair for Colorectal Cancer, King Abdulaziz University, P.O. Box 80205, Jeddah 21589, Saudi Arabia; ^2^Department of Pathology, King Abdulaziz University, P.O. Box 80205, Jeddah 21589, Saudi Arabia; ^3^Center of Excellence in Genomic Medicine Research, King Abdulaziz University, P.O. Box 80205, Jeddah 21589, Saudi Arabia; ^4^Department of Pathology, Faculty of Medicine, Minia University, El Minia, Egypt; ^5^Department of Medicine, King Abdulaziz University, P.O. Box 80205, Jeddah 21589, Saudi Arabia

## Abstract

*Background and Aims*. Villin is a highly specialised protein and is expressed in intestinal and renal proximal tubular epithelium. It was detected in colorectal carcinomas (CRC) and other nongastrointestinal tumours. The aim of the current study is to investigate the immunohistochemical expression of villin in a subset of primary CRC and determine its relation to tumour differentiation, invasion, nodal metastasis, recurrence, and disease-free survival. *Patients and Methods*. Paraffin blocks of 93 cases of CRC were retrieved constituting 93 primary CRC and 58 adjacent normal mucosa. Immunohistochemistry was performed using antivillin antibody. The extent (%) of villin immunoexpression was categorised for statistical analysis. Statistical tests were used to determine the association of villin with clinicopathological characteristics: age, sex, tumour location, tumour size, depth of invasion, tumour grade, nodal metastasis, lymphovascular invasion, margin status, recurrence, and survival. *Results*. Villin immunostaining results showed that villin is downregulated in CRC. Villin has no association with age, sex, tumour location, depth of invasion, nodal metastasis, lymphovascular invasion, margin status, and recurrence. However, villin is expressed in higher rate in CRC less than 5 cm, well- and moderately differentiated CRC. Poor survival was associated with tumour with low villin immunoexpression. *Conclusion*. Villin was downregulated in CRC. Villin immunoexpression in CRC is associated with better survival, well-differentiated tumours, and small-sized tumours. Villin has no significant association with disease recurrence or nodal metastasis. More *in vivo* and *in vitro* studies are required for further elucidation of how villin may be involved in CRC.

## 1. Introduction

Although 70% of colorectal carcinoma (CRC) cases undergo a curative surgery, 50% of surgically cured patients will have at a time an advanced local recurrence or metastases [1]. CRC is a common type of cancer with a considerably poor prognosis and mortality all over the world. The extent of local invasion and tumour metastasis are important factors to determine disease outcome. Distant metastases aggravate treatment failure, and patients will be subjected to palliative treatment. Molecular markers of metastasis are essential to improve treatment protocols [1, 2]. Understanding the molecular pathology underlying CRC needs continuous efforts to discover more prognostic molecules to predict disease outcome and improve therapeutic interventions. The Saudi Arabian National Cancer Registry in 2005 showed that CRC represented 5.3% of all tumours. CRC is the most common malignancy in Saudi males, while in females, CRC is the third most common malignancy and represented 4% of all tumours [3]. 

Villin is a protein that belongs to the gelsolin family of calcium-regulated actin-binding proteins [4]. It was first isolated and characterised in the microvilli of intestinal epithelium [5]. Hence, the name villin came from. Microvilli are located on the apical membrane (brush border) of polarised epithelial cells and increase the surface area for the absorption of nutrients. Microvilli are sustained by bundles of parallel actin filaments that are organised by multiple actin-binding proteins including villin [6]. Villin constitutes a major component of the brush border cytoskeleton. It functions in the capping, severing, and bundling of actin filaments [4, 7]. Villin is a highly specialised protein and specifically expressed in intestinal epithelium and renal proximal tubules. Villin had been detected in CRC and has been used to differentiate neoplasms of intestinal origin from nonintestinal neoplasms [8, 9]. Villin is also expressed in gastrointestinal neuroendocrine tumours [10]. 

As the outcome of CRC is largely dependent on the extent of local and metastatic tumour spread, the identification of molecular markers of metastatic clones is essentially needed for developing new therapies in CRC. The aim of the current study is to investigate the immunohistochemical expression of villin in a subset of primary CRC and determine its relation to tumour differentiation, invasion, nodal metastasis, and recurrence.

## 2. Patients and Methods

The study included 93 cases of CRC (in the period from 2003 to 2009) that were retrieved from the archives of the Department of Pathology at King Abdulaziz University, Jeddah, Saudi Arabia.  Table 1 shows the clinicopathological characteristics of this subset of patients. Age ranges from 24 to 91 years (median 60 years). The study was approved by the Research Committee of the Biomedical Ethics Unit, Faculty of Medicine, King Abdulaziz University. 

### 2.1. Immunostaining of Villin

Four *μ*m thick sections were prepared from paraffin blocks and mounted on positive charged slides (Leica Microsystems Plus Slides, Menzel, Braunschweig, Germany). Sections were deparaffinised in xylene and rehydrated in graded alcohol. Slides were immersed in H_2_O_2_ (0.3%) for 12 minutes to block the endogenous peroxidase activity. Slides were then pretreated in microwave oven in 10 mM citrate buffer (pH 6) for three cycles of 5 minutes each. Anti-human rabbit polyclonal antibody to villin (E1664 from Springer Bioscience) was used at dilution 1 : 50 with incubation time 30 minutes at room temperature. Immunostaining was carried out in an automated immunostainer (BenchMark XT, Ventana Medical systems Inc., Tucson, AZ, USA) according to the manufacturer's instructions. Subsequently, slides were washed, counterstained with Mayer's haematoxylin, and mounted. Negative control (by substitution of primary antibody with Tris-buffered saline) was used. Normal intestinal mucosa was used as an internal positive control.

### 2.2. Interpretation of Villin Immunostaining

In order to evaluate villin immunostaining, we first observed the subcellular localisation of villin in normal colonic mucosa adjacent to tumours and in tumour cells as previously described. Three patterns of positive villin staining were observed: a primary brush border pattern (apical membranous), a primary cytoplasmic pattern, and mixed diffuse cytoplasmic pattern with brush border staining which was also observed [8, 9]. The apical membranous expression and cytoplasmic localisations were scored independently, and the mixed pattern was calculated whenever both patterns were noted together. To assess the extent (%) of villin expression (apical membranous and cytoplasmic), all available normal and tumour cells in each section were counted at microscope magnification of 200x. Positive cells were then counted. The mean values of positivity were calculated and expressed as a percentage of the total number of normal and tumour cells. For statistical analysis, we have categorised the extent of villin immunoexpression as negative expression (0 = 0%), low expression (1 = 1–9%), moderate expression (2 = 10–50%), and high expression (3 ≥ 50%).

### 2.3. Statistical Analysis

Data is presented as the mean ± standard error of the mean (SEM). Differences between two groups of cases for one variable were tested by using the Mann-Whitney test. To test association between three groups of cases for one independent variable, the Kruskal-Wallis test was used. Difference between expression in normal mucosa and tumour was tested by Wilcoxon signed-rank test. Multivariate logistic regression analysis was used to predict lymph node metastasis and recurrence in relation to immunoexpression of villin. Beta coefficient and 95% confidence intervals (CI) were denoted for each analysis. The Kaplan-Meier procedure was used to calculate the survival probabilities, and the log-rank test was used to compare the difference between survivals. The end point for patients was death from tumour (disease-specific survival (DSS)). DSS was calculated as the time from diagnosis to death or to the date of last seen alive. Patients who died of causes other than tumour or unknown causes were censored. Statistical procedures were performed using SPSS Release 16.0. Statistical significance was determined at *P* value of ≤ 0.05, and tests were two sided.

## 3. Results

**Figure 1:**
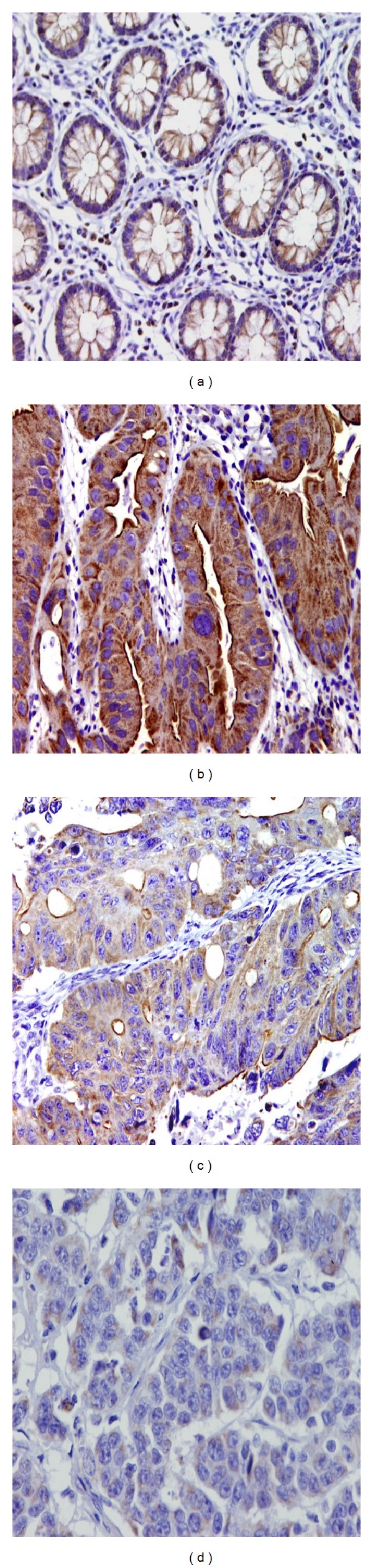
Villin immunoexpression in normal colonic mucosa and in colorectal carcinoma using immunohistochemical labelling with antivillin antibody with diaminobenzidine used as the chromogen and haematoxylin as counterstain. Magnification used was 200x. (a) Villin expression in normal colonic crypts showing a well-organised brush border pattern together with minor cytoplasmic dots in all crypts. (b) Expression in a well-differentiated tumour; a strong staining is shown in both cytoplasm and brush border (mixed pattern). (c) Villin expression in a moderately differentiated colorectal carcinoma; staining is shown in both cytoplasm and brush border (mixed pattern) with less extent and intense staining than in (b). (d) Villin expression in a poorly differentiated colorectal carcinoma showing very minor cytoplasmic staining with no membranous staining.

*Basic Data of Villin Immunostaining.* We have observed a brush border (apical membranous) with minor cytoplasmic component in 100% of samples of normal colonic and intestinal crypts (Figure 1(a)). In tumour cells, cytoplasmic immunoexpression was noted in 84.9% of examined samples. In 60.2% of samples, there was an associated apical membranous staining, mixed pattern (data is shown in Table 2) (Figures 1(b)–1(d)). A high level of villin protein immunoexpression was observed in normal mucosa adjacent to tumour (100% of samples; mean 77.84%; SEM: 3.284) than in tumour samples (84.9% of cases; mean: 17.32%; SEM: 1.514); *P* < 0.001 (data is presented in Table 2). In normal mucosa, the incidence of negative and low categories of expression was not observed, while high expression constitutes a large number and high extent *P* < 0.001. Also in tumour samples, the negative and low expression categories were much lower than moderate category *P* < 0.001 (data is shown in Table 3).

### 3.1. Villin Immunoexpression and Clinicopathological Characteristics

The distribution of villin expression in tumour samples in relation to clinicopathological characteristics is presented in Table 4. There was no statically significant difference in villin immunoexpression in regards to age, sex, tumour location, depth of invasion (primary tumour), nodal metastasis, lymphovascular invasion, margin status, recurrence, or status at end point. However, the mean of villin immunoexpression in well differentiated, and moderately differentiated tumours are higher than in poorly differentiated tumour (*P* = 0.035). Also, the mean of villin immunoexpression was statically higher in tumours measuring less than 5 cm than in larger tumours (*P* = 0.042).

**Figure 2:**
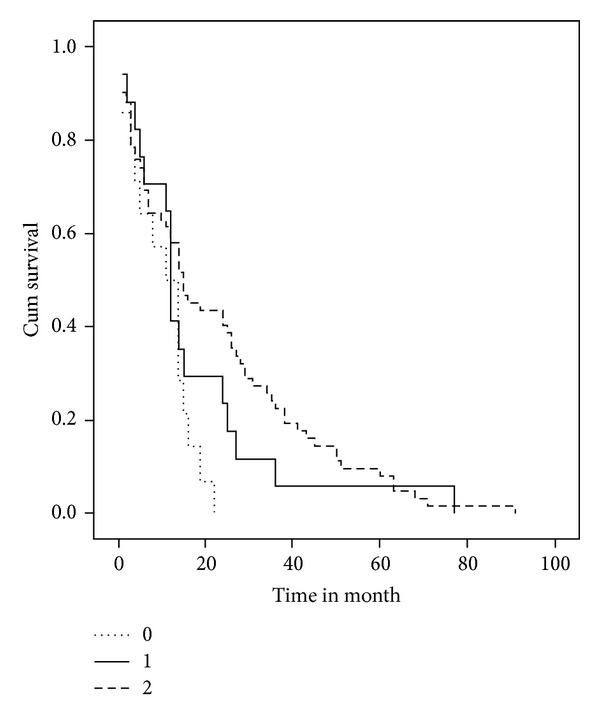
Disease-free survival curve (Kaplan Meier) according to villin immunostaining; 0: negative villin expression; 1: low villin expression; 2: moderate villin expression (log-rank = 6.604, *P* = 0.037).

On multivariate logistic regression analysis, villin immunoexpression showed no statistical significance as an independent predictor of lymph node metastasis (beta coefficient = 1.652, confidence interval = 1.447–1.857, *P* value = 0.255) or of recurrence: (beta coefficient = 1.532, confidence interval = 1.327–1.737, *P* value = 0.488). The Kaplan-Meier survival analyses showed that villin immunoexpression in CRC has a significant association with a more favourable disease-specific survival (log-rank = 6.604, *P* = 0.037) (Figure 2).

## 4. Discussion

CRC has a relatively poor prognosis, in which the outcome is determined by the extent of local and metastatic tumour spread. The 5-year survival rate ranges from 90% in stage I disease to <10% in metastatic disease (stage IV) [1]. The molecular pathogenesis of CRC involves transformation of normal colonic epithelium, adenoma, and then carcinoma. This transformation is tightly linked to molecular alterations that lead to abnormal cellular differentiation, proliferation, migration, and apoptosis [11]. Currently, several prognostic factors exist, including clinical staging classification. However, specific markers for metastatic clones in CRC are required to predict the disease outcome and improve the therapeutic intervention.

Villin is an actin regulatory protein that is localised in all the intestinal epithelial cells and gastrointestinal tract-associated exocrine glands [12, 13]. Villin regulates intestinal epithelial cell morphology, actin reorganisation, and cell motility [14, 15]. Villin expression in malignant tumour was described first by Moll et al. [9] and Bacchi and Gown [8]. Villin has been also detected in hepatocellular carcinoma (9%) and showed cytoplasmic staining pattern. In cholangiocarcinoma expression was detected in 20% of cases and showed only cytoplasmic pattern [16]. In another study, 31% of HCCs were villin positive. Most displayed a cytoplasmic staining pattern. In 22% of cholangiocarcinoma cases there was cytoplasmic and apical staining for villin [17]. The expression of the villin protein has been detected in renal cell carcinoma [18]. Accumulation of villin has been observed at the transcript level in HT29 colon adenocarcinoma cells [19].

In this study, we examined the expression and localisation of villin protein in a subset of CRC and a number of adjacent histologically normal mucosa. As reported before [8], we have described three patterns of cellular localisation of villin: apical membranous (brush border), cytoplasmic, and mixed pattern. In normal mucosa, 100% of examined samples showed villin immunoexpression with brush border localisation and a minor cytoplasmic element. In tumour samples, villin was found to be expressed in 84.9% of cases; cytoplasmic localisation was detected in 100% of positive cases, while the characteristic brush border was detected in 70.9% of positive cases. Arango et al. also observed mislocalisation of villin CRC away from normal brush border localisation [6]. In our study, 29.1% of positive cases were lacking the characteristic brush border staining. Several authors have reported that villin brush border staining is exclusive for gastrointestinal adenocarcinoma [8, 13, 20]. However, villin expression was described in nongastrointestinal carcinomas such as renal cell carcinoma and endometrial carcinoma [9]. Also, abnormal localisation has been described in neuroendocrine carcinoma from different organs [10]. The mechanism of translocation of villin to the cytoplasm of malignant cells has not yet been clarified and needs to be addressed. We may speculate that villin production is required at different stages of epithelial cell transformation in CRC.

In our study, we demonstrated immunoexpression in CRC is downregulated than normal. This is reflected at two levels, the percentage of positive cells are less than normal (84.9%). Second is that in CRC samples majority of positive cases are moderately expressing villin while in normal mucosa the majority showed high villin expression. This finding is consistent with findings of Werling et al. [21] and Arango et al. [6]. However, other investigators stated that villin had been shown to be expressed in almost 100% of primary and metastatic adenocarcinomas of the colon [8, 9, 22, 23]. The difference may be explained by using different sample size, antibody clone, or technical consideration in immunohistochemistry. So, absence of villin expression in a metastatic poorly differentiated CRC does not exclude gastrointestinal origin [6]. Decrease in villin level has been described in pathological conditions of colon such as Crohn's disease and ulcerative colitis [24], and this is supported by finding that villin knockout mice have higher susceptibility to intestinal cell damage [4]. Villin expression is regulated by Cdx-2 transcription factor which enhances villin expression [25]. Cdx-1 (transcriptional factor) is strongly correlated with villin expression. Villin downregulation in CRC is a consequence of loss of Cdx-1 expression; a likely possibility is that multiple proteins that are Cdx-1 targets will be downregulated in parallel and that collectively this results in a loss of glandular differentiation [6].

The relation between villin and clinicopathological characteristics has been addressed in our study. We could not establish association between villin immunoexpression and nodal metastasis. Also villin immunoexpression was not able to predict nodal metastasis. However in other studies, cells expressing villin migrate faster than villin-null cells [15] and villin may be modified during metastasis [7]. In addition, there was no statistically significant relation between villin expression and patients' age, tumour location, depth of invasion, lymphovascular invasion, margins, or recurrence.

 We found also that villin expression was higher in well-differentiated and moderately differentiated CRC than in poorly differentiated CRC, which is similar to what was found previously [6]. Bacchi and Gown showed that no relationship was found between the presence, or pattern of expression, of villin and the state of tumour differentiation [8]. Whether villin is preserved, downregulated, or lost completely in poorly CRC has to be verified by further studies due to small number of poorly differentiated samples in our study and previous studies. 

In this study, villin immunoexpression in tumours with a size less than 5 cm in diameter was significantly higher than in those of 5 cm or more. This finding is genuine and has no comparable results. This means that villin may be involved in inhibiting tumour growth and proliferation. Villin is involved in regulation of actin dynamics, signal transduction, cell morphology, epithelial-to-mesenchymal transition, cell migration, cell invasion and cell survival in renal cell lines, intestinal cell lines, and villin-null mice [14, 15, 26–29]. Another contradictory finding in villin is an epithelial cell-specific antiapoptotic protein [7, 12]. The exact mechanism behind this observation remains a matter for further research including proliferation assay and invasion assay.

We have found that CRC with higher villin immunoexpression are associated with better survival outcome. On the other hand, Arango et al. found no such association between villin staining intensity and overall survival in CRC [6].

Most current studies focus on using villin as a differentiation marker for metastatic gastrointestinal adenocarcinomas. Data is still lacking regarding the biological importance of villin immunoexpression in CRC and other tumours. We need to extend our knowledge in discovering the mechanistic pathways of villin in tumorigenesis. 

Limitations of our study are incomplete follow-up data of some cases and small number of poorly differentiated CRC in samples examined. 

## 5. Conclusion

In summary, villin was downregulated compared to normal counterpart. We have shown that villin immunoexpression in CRC is associated with better survival, well-differentiated tumours, and small-sized tumours. Villin has no significant association with disease recurrence or nodal metastasis. More *in vivo* and *in vitro* studies are required for further elucidation of how villin may be involved in CRC. 

## Figures and Tables

**Table 1:** Clinicopathological characteristics of cases.

Item	Number (%)
Age	
<60 years	45/93 (48.4%)
≥60 years	48/93 (51.6%)
Sex	
Male	44/93 (47.3%)
Female	49/93 (52.7%)
Tumour location	
Right colon	30/93 (32.2%)
Left colon	26/93 (28%)
Rectum	37/93 (39.8%)
Tumour size	
<5 cm	29/93 (31.2%)
≥5 cm	24/93 (25.8%)
Data not applicable	40/93 (43%)
Primary tumour	
T1	0/93 (0%)
T2	12/93 (12.9%)
T3	10/93 (10.8%)
T4	31/93 (33.3%)
Data not applicable	40/93 (43%)
Tumour grade	
Well differentiated	25/93 (26.9%)
Moderately differentiated	63/93 (67.7%)
Poorly differentiated	5/93 (5.4%)
Nodal metastasis	
Positive	31/93 (33.3%)
Negative	24/93 (25.8%)
Data not applicable	38/93 (40.9%)
Lymphovascular invasion	
Positive	7/93 (7.5%)
Negative	46/93 (49.5%)
Data not applicable	40/93 (43%)
Margin status	
Involved	6/93 (6.5%)
Free	47/93 (50.5%)
Data not applicable	40/93 (43%)
Recurrence	
Recurrence	30/93 (32.25%)
No recurrence	33/93 (35.5%)
Data not available	30/93 (32.25%)
Status at end point	
Died of disease	23/93 (24.7%)
Alive	70/93 (75.3%)

T1: tumour invades submucosa; T2: tumour invades muscularis propria; T3: tumour invades through the muscularis propria into the subserosa or into nonperitonealised pericolic or perirectal tissues; T4: tumour directly invades other organs or structures and/or perforates visceral peritoneum.

**Table 2:** The incidence of villin immunolocalisation in CRC and normal mucosa.

	Frequency of expression (positive cases)		Pattern of localisation (number (%))		
	Total	Number (%)	Brush border	Cytoplasmic	Mixed
Adjacent normal colonic mucosa	58	58 (100%)	56 (96.5%)	0 (0%)	2 (3.5%)
CRC	93	79 (84.9%)	0 (0%)	23 (24.7%)	56 (60.2%)

Brush border expression: a primary brush border staining only; cytoplasmic expression: a diffuse cytoplasmic expression without any brush border staining; mixed: a primary cytoplasmic staining pattern with minor brush border staining.

**Table 3:** Category of villin immunoexpression.

Category	Normal adjacent mucosa			Tumours		
	Number (%)	Mean (SEM)	*P* value*	Number (%)	Mean (SEM)	*P* value*
Negative (0)	0/58 (0%)	0 (0)	<0.001	14/93 (15%)	0 (0)	<0.001
Low (1)	0/58 (0%)	0 (0)		17/93 (18.3%)	5.47 (0.73)	
Moderate (2)	7/58 (12.1%)	28.57 (4.59)		62/93 (66.7%)	24.16 (1.61)	
High (3)	51/58 (87.9%)	84.61 (2.46)		0/93 (0%)	0 (0)	

*The Kruskal-Wallis test; SEM: standard error of the mean.

**Table 4:** Distribution of villin-positive immunoexpression in relation to clinicopathological characteristics.

	Number (%)	Mean (SEM)	*P* value
Age			
>60 years	38/45 (84.4%)	16.24 (2.13)	0.558^⊠^
≥60 years	41/48 (85.4%)	18.33 (2.16)	
Sex			
Male	35/44 (79.5%)	15.8 (2.26)	0.270^⊠^
Female	44/49 (89.8%)	18.69 (2.04)	
Tumour location			
Right colon	25/30 (83.3%)	17.6 (2.33)	0.891*
Left colon	21/26 (80.8%)	16.69 (3.16)	
Rectum	34/37 (91.9%)	17.54 (2.51)	
Tumour size			
<5 cm	27/29 (93.1%)	20.21 (2.96)	0.042^⊠^
≥5 cm	17/24 (70.8%)	12.42 (2.38)	
Primary tumour			
T2	10/12 (83.3%)	14.33 (4.303)	0.120*
T3	9/10 (90%)	24.3 (4.41)	
T4	25/31 (80.6%)	15.129 (2.56)	
Tumour grade			
Well differentiated	22/25 (88%)	20.20 (14.99)	0.035*
Moderately differentiated	52/63 (82.5%)	16.95 (14.39)	
Poorly differentiated	3/5 (60%)	3.6 (4.62)	
Nodal metastasis			
Positive	24/31 (77.4%)	14.19 (13.11)	0.229^⊠^
Negative	22/24 (91.7%)	18.63 (15.46)	
Lymphovascular invasion			
Positive	6/7 (85.7%)	12.29 (3.39)	0.729^⊠^
Negative	38/46 (82.6%)	16.913 (2.22)	
Margin status			
Involved	4/6 (66.7%)	20.33 (7.63)	0.651^⊠^
Free	40/47 (85.1%)	15.79 (2.04)	
Recurrence			
Recurrence	27/30 (90%)	19.36 (2.6)	0.474^⊠^
No recurrence	29/33 (87.9%)	16.8 (2.58)	
Status at end point			
Died of disease	21/23 (91.3%)	18.22 (3.11)	0.748^⊠^
Alive	58/70 (82.6%)	16.74 (1.74)	

^⊠^The Mann-Whitney test; *The Kruskal-Wallis test; SEM: standard error of the mean.
